# Optimized Synthesis According to One-Step Process of a Biobased Thermoplastic Polyacetal Derived from Isosorbide

**DOI:** 10.3390/polym8080294

**Published:** 2016-08-10

**Authors:** Nadia Hammami, Nathalie Jarroux, Mike Robitzer, Mustapha Majdoub, Jean-Pierre Habas

**Affiliations:** 1 Institut Charles Gerhardt, CC 1702, Place E. Bataillon, 34095 Montpellier, France; nadia.hammami@etu.umontpellier.fr (N.H.); mike.robitzer@enscm.fr (M.R.); 2 Laboratoire des Interfaces et Matériaux Avancés, Université de Monastir, 5000 Monastir, Tunisia; mustapha.majdoub@fsm.rnu.tn; 3 Laboratoire Analyse et Modélisation pour la Biologie et l'Environnement, UMR 8587, Université d’Evry-Val d’Essonne, 91025 Evry, France; nathalie.jarroux@univ-evry.fr

**Keywords:** isosorbide, biosourced polymer, polyacetal, synthesis optimization, physicochemical properties, spectroscopies, cyclic/linear architectures

## Abstract

This paper describes both the synthesis and characterization of a biobased and non-aromatic polyacetal produced from the reaction between isosorbide and methylene chloride. The reaction was conducted in an aprotic dipolar and harmless solvent using a one-step, fast and economical procedure. The chemical composition of this polymer was investigated using Nuclear Magnetic Resonance and Fourier Transform Infra-Red spectroscopies. The molecular weights were examined by size exclusion chromatography and MALDI-TOF spectrometry. The synthesis conditions (concentration, mixing speed, solvent nature, stoichiometry, addition mode of one reactan) were found to strongly influence both polymer architecture and reaction yield. Under moderated stirring conditions, the polyacetal was characterized by a larger amount of macro-cycles. Inversely, under higher intensity mixing and with an excess of methylene chloride, it was mainly composed of linear chains. In this latter case, the polymeric material presented an amorphous morphology with a glass transition temperature (*T*_g_) close to 55 °C. Its degradation temperature was evaluated to be close to 215 °C using thermogravimetry according to multi-ramp methodology. The chemical approach and the physicochemical properties are valuable in comparison with that characteristic of other isosorbide-based polyacetals.

## 1. Introduction

**Figure 1:**
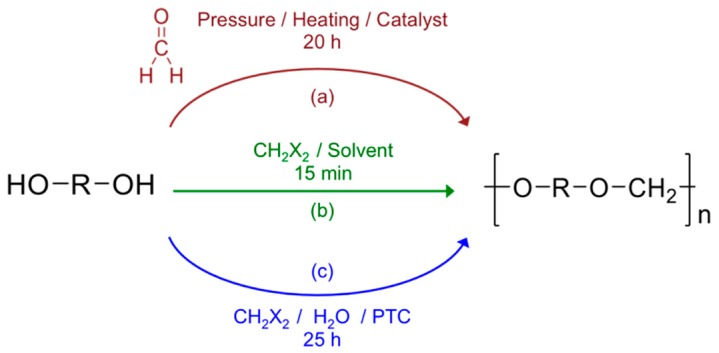
Different routes described in literature for polyacetals synthesis where R is an aliphatic or aromatic sequence and X is given for Cl or Br.

Polyformals (or polyformaldehydes) also known as polyacetals define a polymer category that can be represented by the chemical structure drawn in Figure 1 where R is a divalent aliphatic sequence [1,2,3] or divalent aromatic specie [4,5] including halogenated derivative [4,6,7,8,9]. In the case of aromatic polyacetals, R can contain only one cycle (phenylene) and is eventually substituted as in the case of methylphenylene, dimethylphenylene, chlorophenylene [10]. It can also be made by combination of several aromatic groups either with condensed rings (naphthalene) [11] or conjugated (fluorenyle) [12] or separated by chemical hinges (ether, carbonyl, sulfur, sulfoxide) [4] including short aliphatic sequences C*_n_*H_2*n*_ [10].

Different methods can be used to produce polyacetals. In the case of aliphatic polyacetals, the reaction can be directly performed between aliphatic diols [1,2,13] with formaldehyde or dialkyl formals (route (a)—Figure 1). However, when the diol contains less than 4 carbon atoms, the formation of cyclic structures is favored at the expense of linear macromolecular chains [14]. This chemical approach is not possible in the case of aromatic polyacetals. Indeed, it is well known that the reaction between aromatic diols (i.e., bisphenols) and formaldehyde leads to phenol-formaldehyde resins due to C-alkylation of the phenol instead of the wished O-alkylation [15,16,17,18]. As a consequence, the formal linkage must be produced according to another procedure. One possibility is offered by the mixture and reaction of the bisphenol with methylene halide in a water-immiscible non-polar aprotic solvent in alkaline conditions and in the presence of a phase-transfer catalyst (PTC) such as phosphonium or quaternary ammonium salts (route (c)) [19,20]. The formation of cyclic polyacetals remains possible but seems to be dependent on the experimental conditions such as the reaction temperature or the pressure [5].

**Figure 2:**
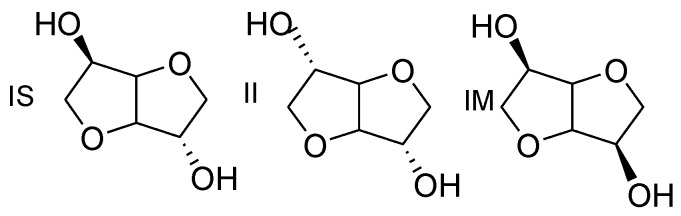
Chemical structures of dianhydrohexitols: isosorbide (IS), isoidide (II) and isomannide (IM).

By applying this methodology, different works investigated the development of polyacetals using bisphenol A (BPA) or its derivatives as possible building block for the R sequence. For instance, Hay et al. reported the reaction of BPA with methylene chloride [10]. It leads to a fully amorphous polymer with a glass transition temperature (*T*_g_) of 85 °C and that is actually made by the mixture of linear chains with cyclic polyacetals. But, to our knowledge, no polyacetal was made using a biobased diphenol/diol whereas the use of harmless building blocks issued from renewable resources is a topic of first interest [21]. Indeed, terms such as “biodegradable”, “biocompatible”, “renewable” and “green” are at the center of many researches devoted to material science in both academic and industrial communities [22]. Actually, biomass offers an important and promising renewable resource for the sustainable production of energy, chemicals and polymeric materials. As one of the most abundant biobased product, isosorbide arouses a real attention in many works [23]. Isosorbide also known as 1,4:3,6-dianhydro-d-sorbitol is obtained from sugar industry by double dehydration of starch derivatives [24,25]. This rigid, chiral, bicyclic and nontoxic molecule was studied in various fields of applications ranging from polymers [26], coating [27,28], surfactant [29,30,31,32], catalysis [33], textile [34], semi finite materials obtained from click chemistry [35,36] (i.e., prepregs) including plasticizers [37,38,39,40] to pharmaceuticals [41,42,43] or biomedical field [44,45]. The difference between isosorbide (IS) in comparison to its two isomers [24] isoidide (II) and isomannide (IM) is the configuration of the two hydroxyl groups as shown in Figure 2.

Isoidide exhibits *exo* and isomannide *endo* configuration for both hydroxyl groups, whereas in isosorbide, the hydroxyl groups in the 2- and 5-position are in *exo* and *endo* configuration respectively [24]. This distinction is of first importance because the *endo* hydroxyl group present in isosorbide is known to present a reduced reactivity, due to the steric hindrance and the intramolecular hydrogen bonding formed with the oxygen atom in the neighboring ring. Actually, this peculiarity is a serious limitation for the synthesis of high molar weight polymers [46,47,48].

In previous publications, 1,4:3,6-dianhydrohexitols were identified to be a reliable and useful biobased chemical to prepare new monomers and a large range of functional polymers (polyesters, polyamides, polycarbonates, polyurethanes) [23]. In this paper, we explored the possible development of a new kind of polyacetals using isosorbide as a possible substituent of BPA. Our polymer named PAIS (for PolyAcetal from Isosorbide) was obtained from the polycondensation reaction between isosorbide with methylene chloride in aprotic dipolar and nontoxic solvent such as dimethylsulfoxide (DMSO) [49]. This synthesis is made according route (b) as represented in Figure 1. Different analytical techniques (NMR spectroscopy, MALDI-TOF spectrometry) were used to characterize its chemical characteristics. Reaction yield, polymer architecture and molar weight were found to be strongly dependent on the synthesis conditions such as the temperature, the mixing speed but also the monomer concentration. After optimization of the reaction parameters, the physicochemical properties of the polymer were carefully evaluated using differential scanning calorimetry (DSC) and thermogravimetry (TGA).

To our knowledge, the most comparable polymer is that described by Rajput et al. with the synthesis of a polymer from the reaction of isosorbide with chloromethyl methyl ether [50]. This polymer is characterized by a chemical skeleton quite close to that specific of our own polyacetal. But as we will show in this paper, our PAIS presents many advantages. First of all, it is based on one-step, fast and cost-effective procedure. Indeed, the reaction can be performed in an aprotic and harmless solvent (DMSO) at ambient temperature, in about 15 min and with a final yield of 80%. In contrast, the polymer described by Rajput et al. is obtained with many synthesis steps. It requires a longer reaction time since 48 h are necessary to complete the reaction even at high temperature (90 °C). Moreover, the reaction yield is slightly lower (71%) than in our own reaction (80%). Finally, it should be noted that the process used in Rajput’s work involves many hazardous solvents (chloromethyl methyl ether, 6 diethyl azodicarboxylate, THF, dimethoxymethane). Rajput’s polymer presented a semi-crystalline morphology since a glass transition of 52 °C and a melting temperature of 117 °C were measured. The onset of the thermal degradation of Rajput’s polymer defined by a weight loss of 10% (i.e., a remaining weight of 90%) was close to 175 °C. We will see that our PAIS is characterized by a higher level of ultimate performances (glass transition temperature, thermostability)

## 2. Materials and Methods

### 2.1. Materials

Isosorbide also named “dianhydro-d-glucitol” or 1,4:3,6-dianhydro-d-sorbitol (CAS number 652-67-5—98%), methylene chloride (CH_2_Cl_2_), potassium hydroxide (KOH), reagent grade solvents i.e., dimethyl sulfoxide (DMSO) and methanol (MeOH) were purchased from Sigma-Aldrich Chimie (Lyon, France). All these chemicals were used without further purification.

### 2.2. Techniques

Nuclear magnetic resonance (NMR) spectra were recorded on a Bruker spectrometer operating at 300 MHz (^1^H) and 75 MHz (^13^C) at room temperature with CDCl_3_ and DMSO-d_6_ as the solvent. Chemical shifts (^1^H NMR) were referenced to the peak of residual CHCl_3_ at 7.26 ppm. Chemical shifts (^13^C NMR) were referenced to DMSO-d_6_ at 39.5 ppm [51,52]. This equipment used an Inversegate sequence with long D1 (>60 s) that made it possible the registering of ^13^C NMR quantitative analyses. The calibration of the apparatus was previously checked by registering the signal characteristic of a model compound (8-aminooctanoic acid) and with integration of the corresponding carbonyl bands.

FT-IR spectra were acquired at 25 °C on a Perkin Elmer Spectrum 100 spectrometer equipped with an Attenuated Total Reflectance option (ZnSe crystal). 

Size Exclusion Chromatography (SEC) was performed on a Spectra-Physics apparatus equipped with a Shodex refractive index detector. An analytical column of Styragel HR4E from Waters^©^ (Milford, CT, USA) was used at 60 °C with 0.3 mL·min^−1^ flow rate of DMF calibrated using poly(ethylene oxide) standards. A low concentration of polymer was used in solution (1 mg·mL^−1^) to limit the production of secondary phenomena such as absorption. Non-quantitative MALDI-TOF analyses were performed using a MALDI-TOF Bruker Ultra-Flex TOF/TOF (Bremen, Germany) equipped with a nitrogen laser (337 nm, 40 ns) and a detector. Peptide mixtures were used for external calibration. The ions were accelerated with a potential of 25 kV. The measurements were performed in positive mode POS. The spectra were obtained with 2,5-dihydroxybenzoic acid (DHB) as matrix and NaI and/or KI as ionizing salt. A better signal was usually registered by the combination of both Na^+^ and K^+^ cations. It was not the simple combination of each signal registered with each separate cation especially for highest *m*/*z* values.

The thermo-oxidative stability of the polymer was examined using a Q50 thermogravimetric analyzer (TGA) from TA Instruments^®^ (New Castle, DE, USA). The experiments consisted in registering the weight loss of the sample under air flow (25 mL·min^−1^) as a function of temperature from ambient up to 800 °C. An original method was used to evaluate the real degradation temperature by registering different experiments with specific values of heating rate β ranging from 1 to 20 °C·min^−1^. For each temperature ramp, the “apparent” degradation onset *T*_deg_ was defined at the temperature where the sample residual weight was about 95% of the initial value. The “real” onset of the thermal degradation is evaluated by extrapolation of the curve of *T*_deg_ = *f*(β) to β = 0.

Differential scanning calorimetric experiment (DSC) were registered using a Mettler Toledo DSC-1 (Greifensee, Switzerland), under air and with a heating rate of 5 °C·min^−1^. Before analysis, the sample was cooled down from ambient to 0 °C with a ramp of −3°C/min and maintained at this constant temperature during 15 min to insure thermal equilibrium.

### 2.3. Synthesis

The synthesis of the isosorbide-based polyacetals was explored according to two distinct pathways characterized by specific mixing method. In the first method, a magnetic stirring was used for the mixing step. In the second one, the dispersion of the reaction mixture was insured by a homogenizer Ultra-Turrax T 25 Blender Basic from Ika (Staufen, Germany) equipped with a S25N 25F geometry. In both cases, the composition of the reaction mixture was the same: it consisted of isosorbide (40 mmol), potassium hydroxide powder with a small excess (1.16 equivalent) and DMSO (20 mL). After 5 min of vigorous stirring performed at ambient temperature, an excess of methylene chloride was quickly added in one step to the solution using a syringe. The polycondensation was found to be exothermic since after 10 min of reaction, the temperature was registered to be close to 90 °C. These characteristics did not make it easy to control the temperature during the reaction but no thermal run-away was observed. No synthesis trial was operated for an initial temperature lower than *T* = 19 °C due to the vicinity of DMSO crystallization. In a similar way, the initial reaction temperature was chosen as lower than *T* = 45 °C to limit methylene chloride vaporization before reaction step. 

Whatever the procedure used, the reactive mixture did not present the same aspect depending on the technique employed for the mixing step. Actually, the use of magnetic stirring did not allow a homogenous solution shearing even at maximum speed and the isosorbide salt formed during the synthesis was observed to precipitate. But, only oligomers could be detected even after 2 h of reaction. Finally, the polymer was isolated after 8 h with very low yield going up to 30%. The Ultra-Turrax made it possible the production of higher shearing rate in the reactive mixture. After CH_2_Cl_2_ addition, the solution viscosity strongly increased in spite of the concomitant temperature augmentation. However, the high intensity mixing increased the exchange surface between the isosorbide salt and methylene chloride, thus aiding its dissolution. In other words, it provided a better homogenization in the reaction media. The reaction was stopped after 15 min due to the solidification of the mixture. After cooling, the mixture was diluted with methylene chloride before filtration step to eliminate both KOH excess and KCl formed. The resulting polyacetals named PAIS was precipitated and washed three times in methanol. The polymer was dried overnight in vacuum to give a fibrous, white product with a good yield (60%). Another low molecular weight compound (10%–20%) was obtained by the concentration of the filtrate and precipitation in a mixture of methanol-diethyl ether (25:75). This fraction could be identified by MALDI-TOF as only made of oligomers (see Appendix). 

## 3. Results and Discussion

### 3.1. Reaction Yield

**Figure 3:**
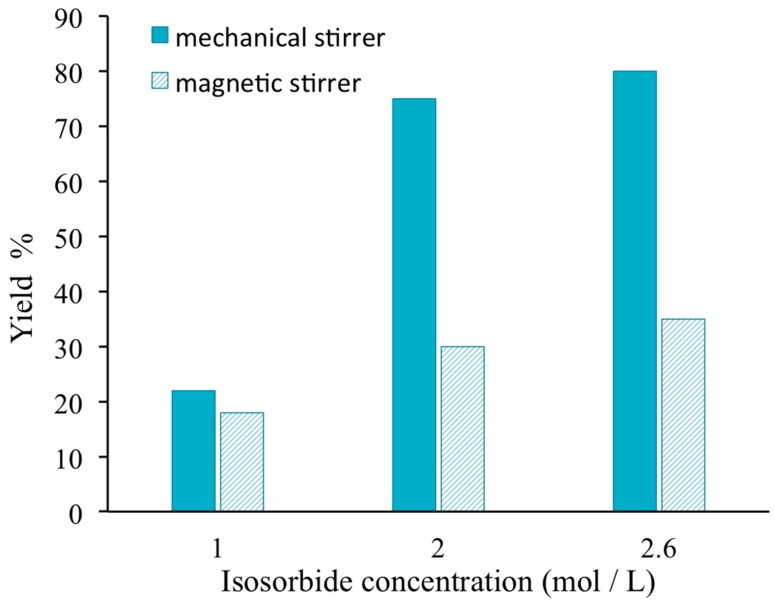
Influence of the stirring method on the reaction yield between isosorbide and methylene chloride.

Figure 3 shows that whatever the initial concentration of isosorbide, the yield reaction is always higher when a mechanical stirrer is used for the mixing of the reactive solution instead of magnetic stirring. The discrepancy between both kinds of stirring is quite important for the highest concentrations due to the mixing difficulty when using a simple magnetic stirring.

### 3.2. FT-IR Analyses

**Figure 4:**
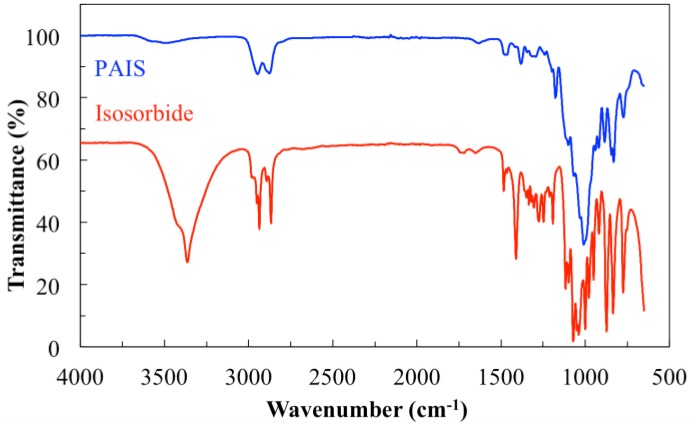
Comparison of FTIR spectra of PAIS and original IS (shifted along vertical axis for easier identification).

The first experiment consisted of the FT-IR characterization of PolyAcetalIsoSorbide (PAIS) polymer obtained by the high-speed stirring method. The resulting spectrum is shown in Figure 4. For clarity, the FT-IR data specific of isosorbide (IS) are also given for better discussion. The comparison of both spectra clearly shows that the PAIS chemical skeleton is based on isosorbide building block as expected. Indeed, PAIS spectrum includes the main bands of the signal characteristic of the IS compound.

FT-IR (cm^−1^) 3466 (terminal OH stretching), 2881, 2945 (CH_2_ stretching), 1018, 1041, 1117 (C–O or C–H stretching). 

**Figure 5:**
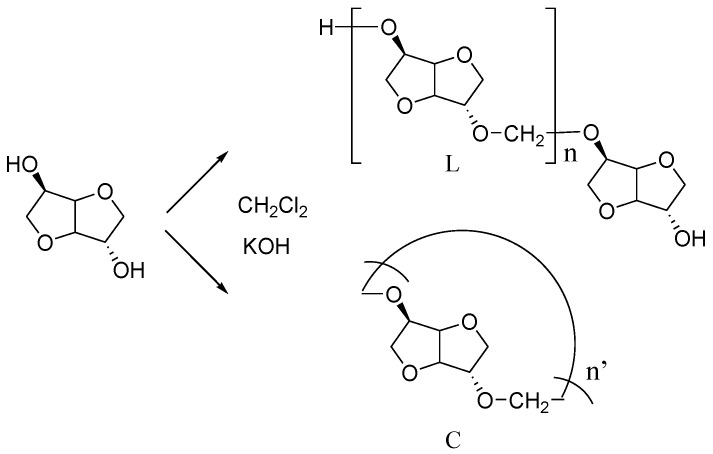
Synthesis of polyacetal obtained from the reaction between isosorbide and methylene chloride. L: Linear structure of PAIS; C: Circular structure of PAIS.

In particular, the intensity of the bands characteristic of the OH groups is quite reduced. This result was expected since in linear chains, the hydroxyl functions are only present in terminal position whereas they do not exist in cyclic structure (Figure 5). In other words, this information shows that the polymerization is successful. But it is not possible to evaluate the information from this if our PAIS contains cyclic structures; and if so, according to which ratio.

### 3.3. ^1^H and ^13^C NMR Experiments

**Figure 6:**
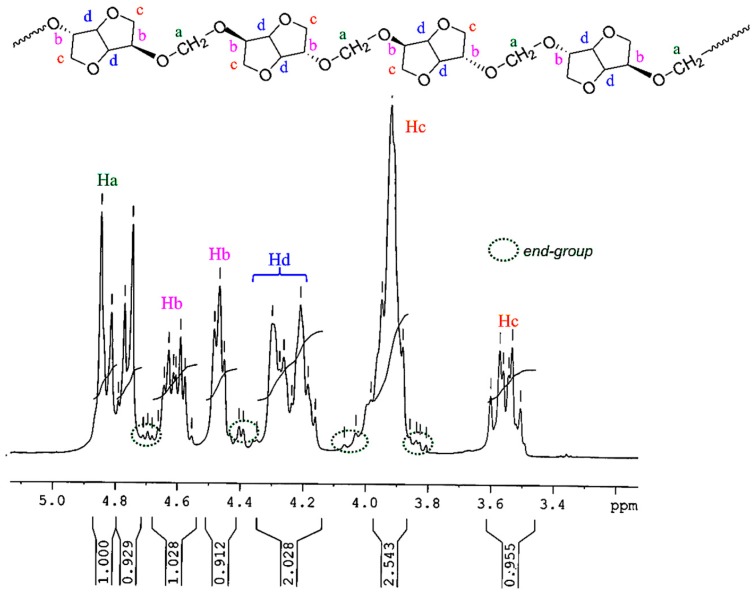
^1^H NMR spectrum of PAIS based on isosorbide, registered in CDCl_3_.

The chemical structure of PAIS produced from high stirring method was investigated by ^1^H NMR technique. The corresponding spectrum is shown in Figure 6. Its integrations agree with the expected structure. Indeed, the ^1^H NMR spectrum of polyacetals exhibits the signals characteristic of the protons present in monomeric structures (a–d). The isosorbide end groups can also be distinguished in Figure 6 by ellipsoidal forms with dot lines.

^1^H NMR (300 MHz, CDCl_3_, 298 K, δ ppm) 4.91–4.72 (m, 2H, Ha), 4.69–4.56 (m, 1H, Hb), 4.52–4.45 (m, 1H, Hb) 4.35–4.16 (m, 2H, Hd) 4.10–3.80(m, 3H, Hc) 3.62–3.50 (m, 1H, Hc).

**Figure 7:**
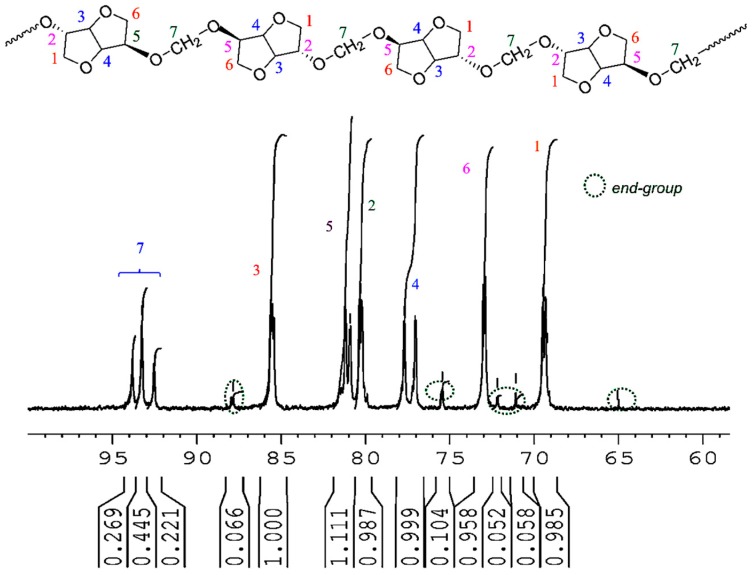
^13^C NMR spectrum of PAIS based on isosorbide as registered in DMSO-d_6_.

The most interesting result is brought with the methylene group present in PAIS polymer. Its presence is detected in Figure 6 by a signal made by multiple bands comprised between 4.91 and 4.72 ppm (peaks a). This peculiarity can be interpreted as the consequence of the different stereoisomerizations induced by *endo* and *exo* links. Further information can be obtained using the quantitative ^13^C NMR technique. Indeed, the corresponding spectrum shown in Figure 7 reveals the presence of three peaks ranging from 94.1 to 92.2 characteristic of CH_2_ groups.

^13^C NMR (75 MHz, DMSO-d_6_, 298 K, δ ppm): 94.1–92.2 (t, C_7_), 86.0–85.2 (m, C_3_) 81.4–80.8 (d, C_5_), 80.6–79.8 (m, C_2_), 78.0–76.8 (d, C_4_), 73.3–72.7 (m, C_6_), 70.0–69.0 (m, C_1_).

**Figure 8:**
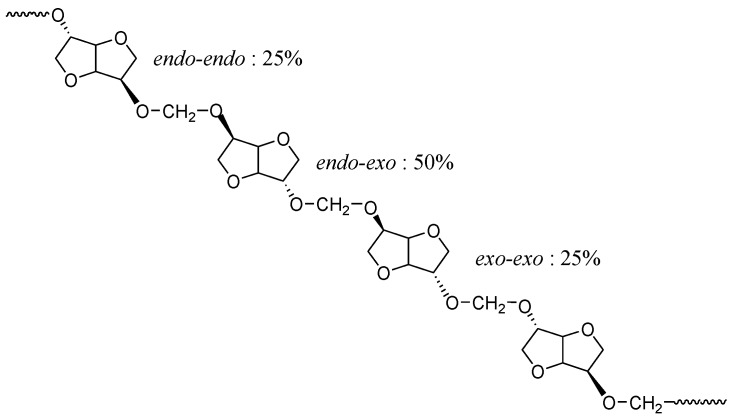
Example of representation of chemical structure of polyacetal based on isosorbide.

This signal pattern results from a random sequence of *exo-exo* (25%), *endo-exo* (50%) and *endo-endo* (25%) connected methylene groups respectively, as shown in Figure 8. These results are supported by previous data reported in literature with polycarbonates of isosorbide [50,53].

### 3.4. MALDI-TOF Analyses

During the synthesis step, we could note that the choice of stirring method was initially quite important to operate in good conditions for the reaction between isosorbide and methylene chloride. More particularly, the process performed under the high-speed blender method gave considerably better results than the “magnetic stirrer method”. Then, MALDI-TOF analyses were registered with each kind of polymer issued from a specific synthesis method to investigate at the macromolecular scale, possible discrepancies existing between the corresponding polymeric architectures. These analyses provide precise information about the types of end-groups present in this new biobased polymer. Indeed, they reveal two distributions of ions (with a repeating unit of 158 g·mol^−1^ which exactly matches the theoretical molar mass of the repeating block of PAIS). Cationized either by sodium or potassium to form (M + Na)^+^ and (M + K)^+^, both signals can respectively be attributed either to linear (L) or cyclic (C) structures as represented in Figure 5. In other words, their respective position along the *m*/*z* scale is specific of a precise polymerization degree (*n* or *n*’) and a macromolecular conformation (L or C). Considering growing *m*/*z* values, the peaks are likely to appear according to the following hierarchy: Na^+^–L_n_, Na^+^–C_n’_, K^+^–L_n_ and finally K^+^–C_n’_ in which *n*’ = *n*+1. Assuming that for close *m*/*z* values, linear and cyclic chains absorb with similar intensity, it is possible to discriminate the relative proportion between each species. Nevertheless, it does not seem reasonable to consider that the polymer absorption coefficient is constant whatever *m*/*z* value. In other words, MALDI-TOF was considered here as a semi-quantitative technique [27]. 

**Figure 9:**
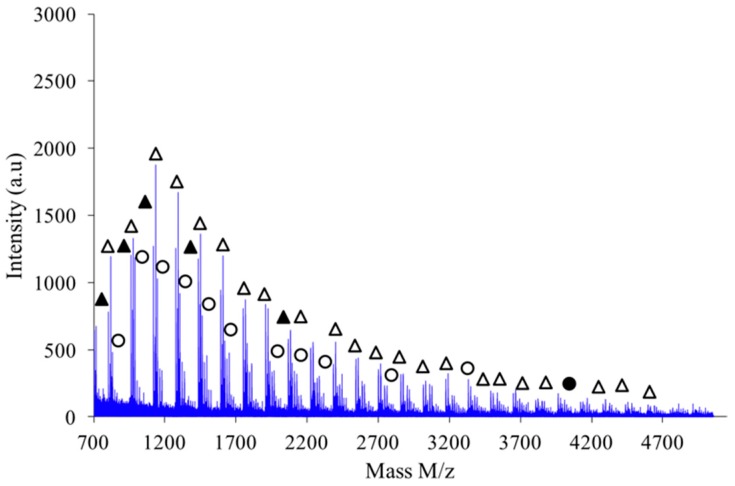
MALDI-TOF mass spectrum of PAIS (*C* = 2 M) prepared under high intensity mixing. Linear chains cationized with K^+^ or Na^+^ are represented by △ and ▲ symbols, respectively. Cyclic species cationized with K^+^ or Na^+^ are depicted by Ο and ●, respectively.

Figure 9 shows the MALDI-TOF registered for PAIS prepared under high intensity mixing and for an initial isosorbide concentration of 2 M. The attribution of the peaks was performed by direct comparison of experimental data with theoretical *m*/*z* values calculated for L or C structures. The corresponding data reveals that the proportion of linear chains is much higher than with low speed stirring conditions. Moreover, the proportion between linear and cyclic chains was also found to be dependent on the monomers concentration.

**Figure 10:**
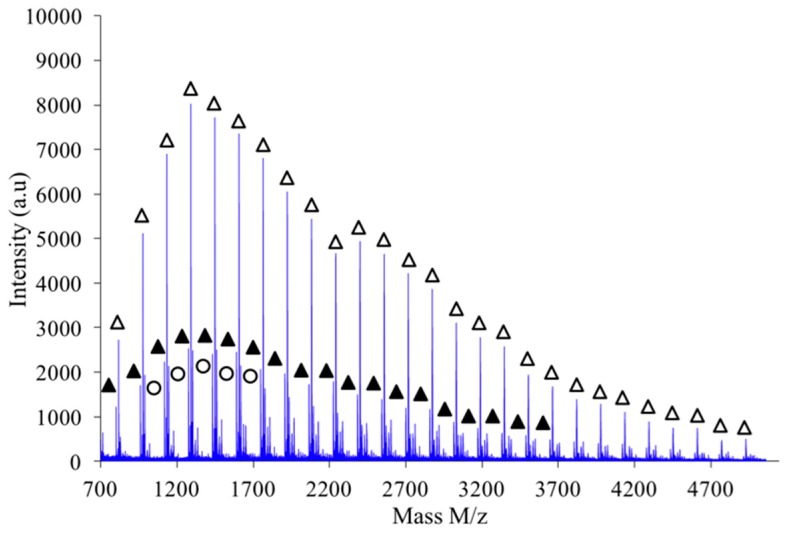
MALDI-TOF mass spectrum of PAIS prepared under high intensity mixing and with high concentration (*C* = 2.6 M). The attribution of the symbols is unchanged compared to Figure 9.

As shown in Figure 10, the polymer obtained by combination of high intensity mixing and high concentration (2.6 M) is almost exclusively made of linear chains L.

**Figure 11:**
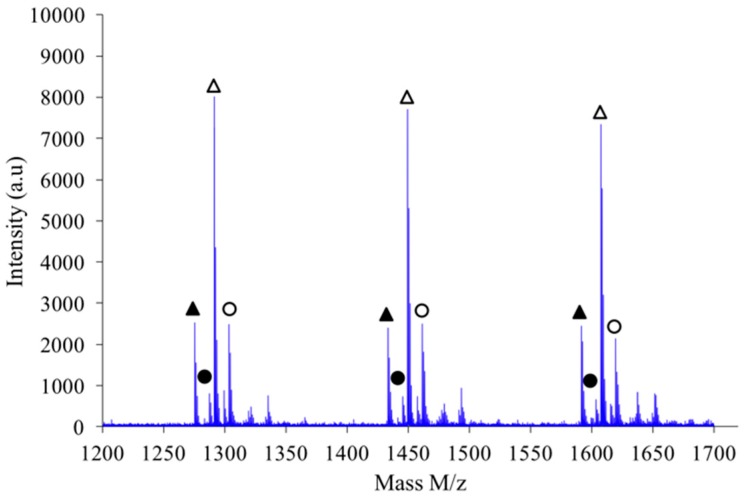
Focus on MALDI-TOF mass spectrum of PAIS prepared under high intensity mixing (2.6 M). Linear chains cationized with K^+^ or Na^+^ are represented by △ and ▲ symbols, respectively. Cyclic species cationized with K^+^ or Na^+^ are depicted by Ο and ●, respectively.

If a focus is made for instance in the *m*/*z* scale ranging from 1200 to 1700 as shown in Figure 11, one notices that the expected hierarchy between cationized species, as introduced before, is checked. Indeed, considering growing *m*/*z* values the peaks appear according to the following hierarchy: Na^+^L_n_, Na^+^–C_n’_, K^+^–L_n_ and finally K^+^–C_n’_ in which *n*’ = *n*+1. Similar classification was obtained with the other synthesis conditions previously described.

**Figure 12:**
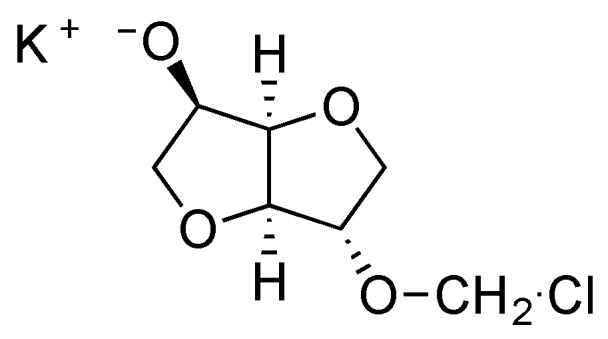
Representation of chemical structure of chloromethyl ether isosorbide intermediate.

All our experimental findings agree with observations previously made in literature with the syntheses of polyacetal derived from bisphenols [5]. Different elements can be proposed to explain the influence of both concentration and stirring rate on the ratio between linear and cyclic chains. The first one is based on the reactivity of the first intermediate formed during the first stage of the reaction between isosorbide and methylene chloride. The chloromethyl ether isosorbide has much higher reactivity than the isosorbide monomer. Thus under mild stirring conditions, the isosorbide salt slowly goes into solution and preferentially reacts with the very reactive chloromethyl ether groups, in preference to reaction with methylene chloride to give reactive chloromethyl intermediates (Figure 12). Under high intensity mixing conditions, the salt dissolution is easier and consequently, its concentration is higher in the reactive mixture. Then chloromethyl intermediates are likely to react with the salt of isosorbide to give linear chains instead of producing cyclization. The same explanation can be retained to justify the influence of the initial monomeric concentration.

These elements are in agreement with Kricheldorf’s theory as regards the kinetically controlled polymerization with chain growth at any concentration and any stage of the polycondensation [54,55]. Indeed, this theory underlines that if side reactions are absent, the chain growth is limited by chain cyclization. As an intermediate conclusion, one can keep in mind that a high stirring rate and/or high isosorbide concentration favor the formation of linear polyacetal chains.

### 3.5. SEC Analyses

**Figure 13:**
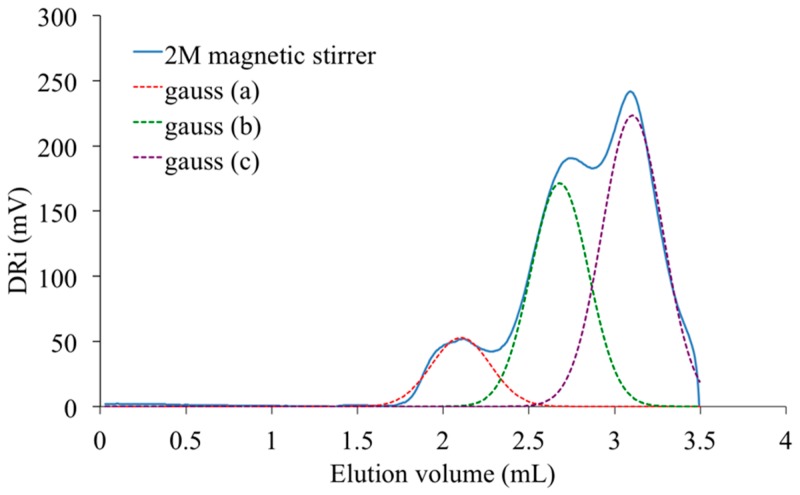
SEC analysis of PAIS synthesized for *C* = 2 M under magnetic stirring. Dashed line: deconvolution based on three individual Gaussian distributions.

SEC experiments were performed to evaluate the molar weight distributions characteristic of different grades of PAIS, that is to say, obtained with different synthesis conditions. Figure 13 proposes as an example, the analysis registered with the PAIS prepared with a constant initial concentration of isosorbide (*C* = 2 M) and under magnetic stirring. The resulting SEC curve seems to be made of three principal peaks that can be distinguished using a deconvolution program based on Gaussian distribution. The corresponding material is made by chemical species with very different molecular weights.

**Figure 14:**
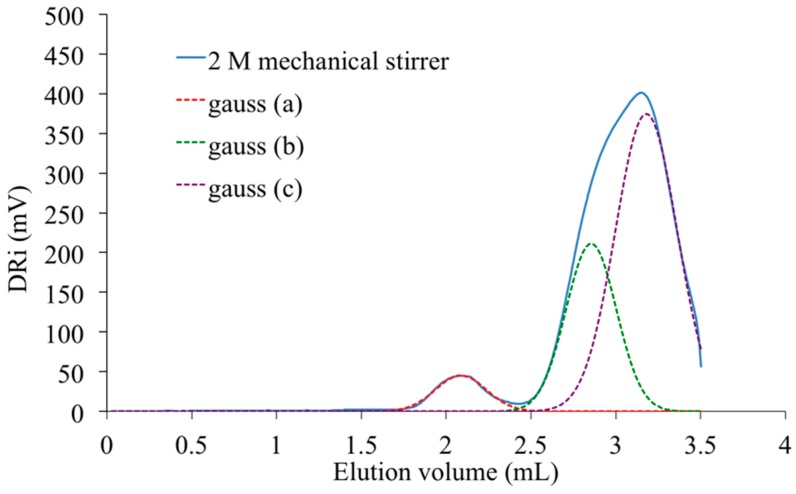
SEC analysis of PAIS synthesized for *C* = 2M under mechanical stirring. Dashed line: deconvolution based on three individual Gaussian distributions.

The peak present at the highest elution volume and centered at 3.1 mL is specific of chemical units with low molar mass. As it is also detected in the signal registered with the PAIS synthesized under mechanical stirring (Figure 14), it is likely attributable to both cyclic and linear chains of low size and that are hardly discriminable. Inversely, the peak registered at 2.1 mL is characteristic of the biggest macromolecular chains present in PAIS. Considering the exploitation of MALDI-TOF analyses, these chains are mainly under cyclic form.

**Figure 15:**
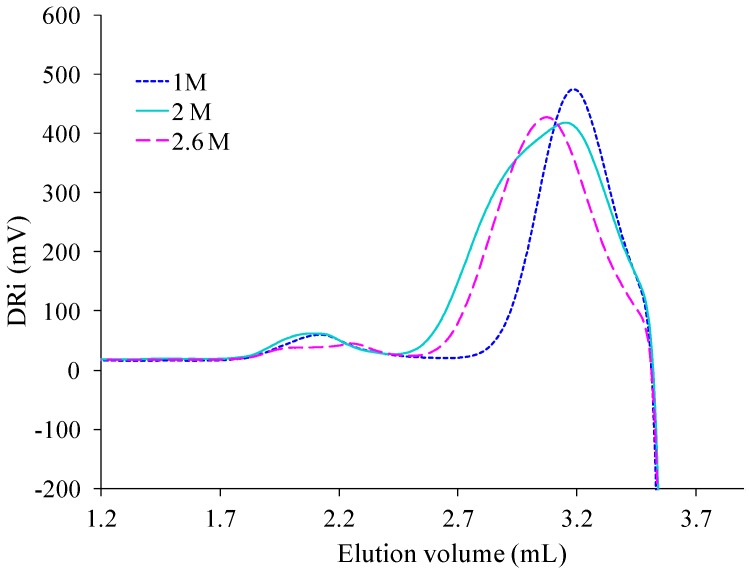
Influence of the initial concentration of isosorbide on the SEC analysis of PAIS.

One can observe the presence of an intermediate peak on both previous SEC signals. It appears as individual and centered at 2.6 mL on the analysis registered for PAIS under magnetic stirring (Figure 13). It is rather a shoulder in the signal registered with the polymer synthesized under high shearing speed (Figure 14). The deconvolution of the corresponding analysis shows that it is more precisely centered at 2.8 mL (Figure 15). Using once more the MALDI-TOF results, the former (2.6 mL) is attributed to cyclic chains, while the latter (2.8 mL) is interpreted as being specific of linear chains, both being characterized by medium size.

SEC technique was retained to complete the evaluation of effects produced by the initial concentration of isosorbide in the reactive mixture on PAIS mass distribution after synthesis under high stirring conditions. The corresponding results are presented in Figure 15. 

**Figure 16:**
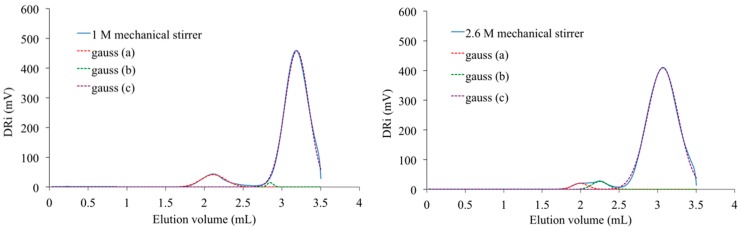
Deconvolution of SEC analyses of PAIS prepared with *C* = 1 M (**left**) and *C* = 2.6 M (**right**).

Its analysis after deconvolution confirms the different attributions of the peaks that were proposed here above. Indeed, the lowest concentration (*C* = 1 M) induced the production of small macromolecular chains characterized by the presence of a peak at highest elution volume (Figure 16). At this concentration, some cyclic structures with high molecular weight are also detected by the existence of a small peak centered at 2.1 mL. The peak characteristic of medium size chains is almost absent. For *C* = 2 M, the SEC signal still shows the same peaks. But, as said before, it also presents a shoulder representative of linear chains with medium molecular weight (Figure 14). At the highest concentration used for PAIS synthesis (*C* = 2.6 M), the complete SEC signal is shifted to lower elution volume revealing the production of linear polymeric chains of higher molar mass (Figure 16). Moreover, the peak that is specific of cyclic chains with high molar mass (2.0 mL) presents quite a reduced amplitude and a little one that is likely due to linear chains of bigger size and is noted at 2.3 mL.

The calibration of the equipment used poly(ethylene oxide) standard as a reference what made it possible the estimation of both molar masses. The average number molar mass was *M*_n_ = 8.63 × 10^3^ g·mol^−1^ while the average weight molar mass was found to be *M*_w_ = 13 × 10^3^ g·mol^−1^. Consequently, the value of the polydispersity index defined as PDI = *M*_w_/*M*_n_ was evaluated close to 1.5. These latter values are slightly higher than the data registered by MALDI-TOF experiments. Indeed, by MALDI-TOF, the highest molar mass was found close to 5000 g·mol^−1^. But one has to keep in mind that the MALDI-TOF experiments do not give any quantitative information about the molecular weight distribution [27]. Furthermore, each technique has its own limited experimental sensitivity. Ultimately, SEC technique used poly(ethylene oxide) as internal reference and this choice is likely to contribute to the discrepancy between MALDI-TOF and SEC data. It is well known that viscosimetry can generally be used to evaluate the molar weight of a polymer from intrinsic viscosity. But in our case, this technique has not been tested because the different exponents used in Mark-Houwink formula were not known due to the newness of our PAIS. The use of SEC equipped with three detectors (refractive index, viscosimetry and light scattering) will be investigated in the near future in order to measure absolute molecular weight. Since linear chains are the only ones that are able to produce entanglements synonym of higher ultimate performances, the highest isosorbide concentration (2.6 M) and high speed were chosen to produce bulk materials for further experimental characterization.

### 3.6. Thermal Analyses

**Figure 17:**
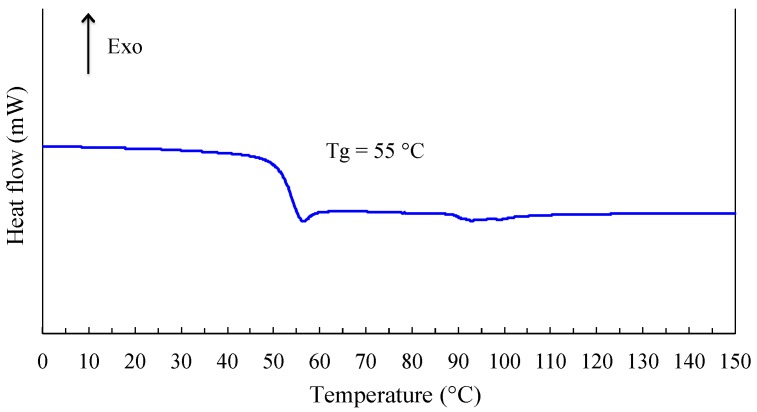
DSC thermogram of linear PAIS produced from the synthesis between isosorbide (*C* = 2.6 M) and methylene chloride using high-speed stirring.

Different physicochemical analyses experiments were performed to evaluate the macroscopic properties of linear polyacetals derived from the reaction between isosorbide and methylene chloride. First experiments were conducted using DSC technique in dynamic mode. The corresponding thermogram presented in Figure 17, only shows a discontinuity in the whole temperature range investigated. Moreover, the registering of the DSC signal during cooling does not show any crystallization. Such information reveals that our polyacetal is an amorphous polymer. Please note that the experimental conditions used cannot be considered as responsible for this characteristic. The glass transition temperature evaluated at the middle of the signal discontinuity is about 55 °C. This morphology is likely due to the asymmetry of hydroxyl groups in isosorbide. In other words, the respective positions of *endo* and *exo* hydroxyl groups induce the formation of stereo irregular polymer. The presence of a little endothermic zone can also be observed for *T* > 90 °C and is likely due to the departure (vaporization) of a quite reduced quantity of water trapped by the polymeric matrix. This hypothesis is supported by the fact that this phenomenon is not registered during a second run.

**Figure 18:**
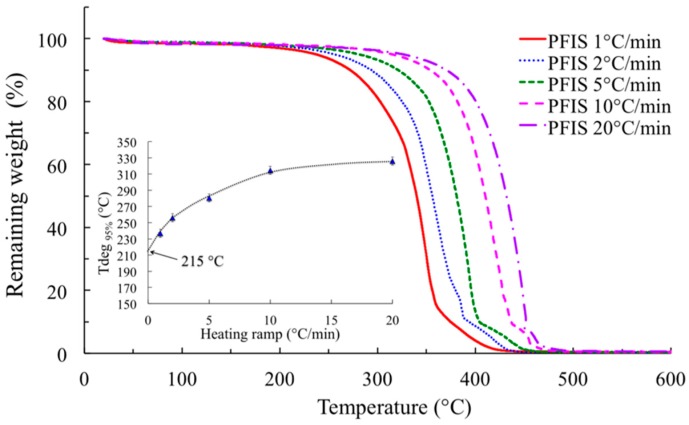
Influence of the heating ramp on the PAIS thermogravimetric profile recorded from 0 to 600 °C under air. Insert: evaluation of the real degradation temperature (the dotted curve is given as a guide).

The thermostability of the same polymer was investigated using thermogravimetry in air atmosphere. The weight loss value of 5% was considered as reasonable to evaluate the onset of the PAIS thermal degradation. At the temperature ramp β of 10 °C·min^−1^ classically taken in TGA analyses, the temperature degradation *T*_deg_ seems to be close to 314 °C for a weight loss of 5% (Figure 18). Actually, this latter value is strongly dependent on the heating rate used in the thermogravimetric analyses. A reduction of the temperature ramp β, synonym of a higher thermal stay of the sample in the oven, induces a shift of the “apparent” *T*_deg_ to lower values. This evolution shows that the classical method based on an only experiment usually performed at 10 °C/min has no sense. Indeed, if the experiment is operated at lower heating ramp, the degradation occurs at lower temperature because the time factor has stronger influence. At the extreme limit, it should be better to perform the analysis at quite reduced heating ramp (i.e., 0.1 °C/min) to take into consideration both time and temperature effects on the degradation process. But, the corresponding experiment is quite long. Nevertheless, by extrapolating the curve of *T*_deg_ = *f*(β) to β = 0, it is possible to evaluate the “real” thermal degradation temperature. Indeed, in these conditions, the degradation process fully takes into account the influence of both temperature and kinetic factors and consequently, the real degradation temperature *T*_deg_ is about 215 °C (insert in Figure 18).

As detailed in the introductory part, the polymer synthesized by Rajput degrades itself for temperature higher than 175 °C considering a weight loss of 10%. With the same weight criteria, our PAIS presents an apparent thermostability up to 353 °C. This hierarchy is likely due to discrepancies in the respective molecular weights of these polymers.

## 4. Conclusions

Our linear polyacetal derived from isosorbide presents up to now a reduced molar weight (*M*_w_ < 15,000 g·mol^−1^). Our synthesis methodology may still appear perfectible but it is important to note that other changes in the PAIS preparation procedure were explored (See Appendix). Further research will be commenced the near future in order to examine the potential use of linear PAIS as chemical intermediates for the synthesis of another polymer. For example, as our linear PAIS is functionalized by two terminal hydroxyl groups, its reaction with biosourced polyacids or anhydride molecules will be carefully studied. Considering the physicochemical properties and structural characteristics of the PAIS developed here, different applications seem possible for this kind of polymer. Its low molecular weight and low *T*_g_ value support a plausible valorization as plasticizer. It is of note that this function has already been satisfied in the past with several generations of aliphatic polyacetal [13] or more recently with low molecular weight polyether [56]. Such an application will first require the characterization of the macroscopic properties of the materials in solid and molten state by dynamic rheometry and mechanical equipment.

## Figures and Tables

**Figure A1:**
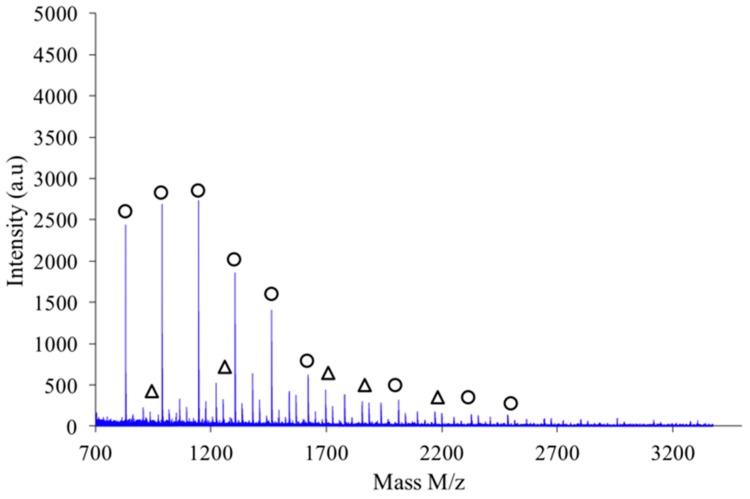
MALDI-TOF mass spectrum of PAIS (*C* = 1 M) prepared under magnetic mixing. Linear chains are represented by triangular symbols (△) while cyclic species are characterized by circular symbols (Ο), both cationized with K^+^ respectively.

**Figure A2:**
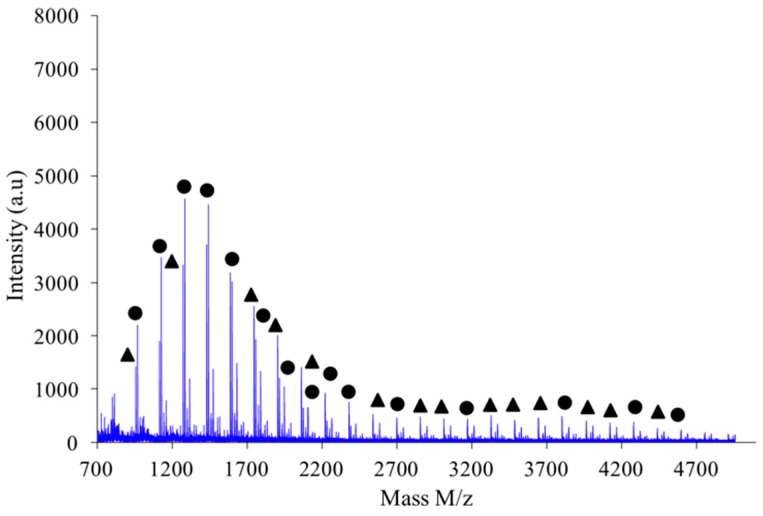
MALDI-TOF mass spectrum of PAIS prepared under magnetic stirring conditions (*C* = 2 M). Linear chains are represented by triangular symbols (▲) whereas cyclic species are characterized by circular symbols (●), both being cationized with Na^+^.

**Figure A3:**
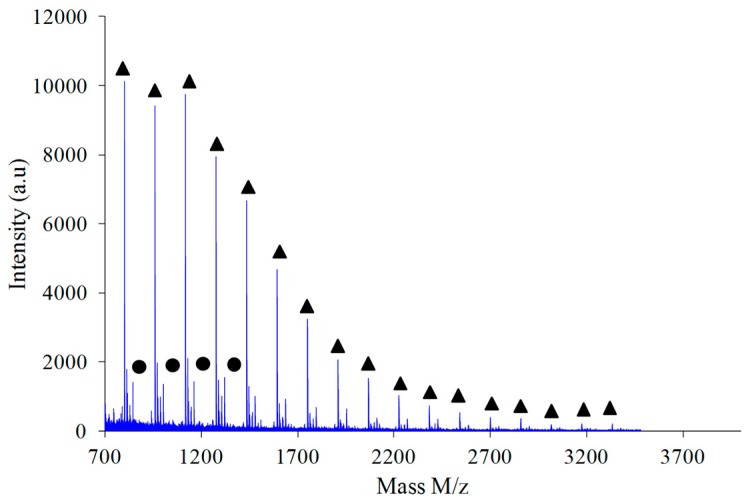
MALDI-TOF mass spectrum of PAIS prepared from the reaction of IS (*C* = 2.6 M) with CH2Cl2 added by drop by drop method and under high intensity mixing. Linear chains are represented by triangular symbols (▲) whereas cyclic species are characterized by circular symbols (●) both being cationized with Na^+^.

**Figure A4:**
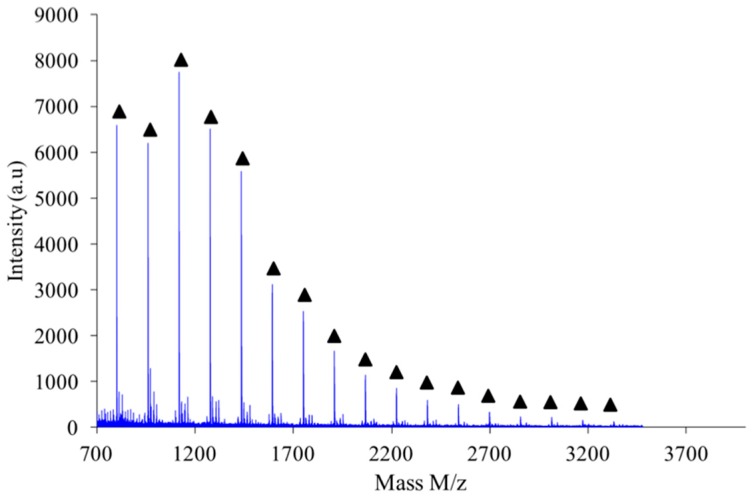
MALDI-TOF mass spectrum of PAIS prepared under high intensity mixing with IS (*C* = 2.6 M) and CH_2_Cl_2_ in stoichiometric proportions. Linear chains cationized with Na^+^ are represented by triangular symbols.

## Abbreviations

The following abbreviations are used in this manuscript:

BPA	Bisphenol A
DHB	2,5-dihydroxybenzoic acid
DSC	Differential Scanning calorimetry
DMSO	Dimethyl Sulfoxide
FT-IR	Fourier Transform Infra-Red
II	Isoidide
IM	Isomannide
IS	Isosorbide
MALDI-TOF	Matrix Assisted Laser Desorption/Ionization Time of Flight
MeOH	Methanol
NMR	Nuclear Magnetic Resonance
PAIS	PolyAcetal (from) Isosorbide
PDI	PolyDispersity Index
PTC	Phase-Transfer Catalyst
SEC	Size Exclusion Chromatography
*T*_g_	Glass transition temperature
TGA	Thermogravimetric Analysis
THF	TetraHydroFuran

## Appendix

First synthesis trials were performed using magnetic stirring. The spectrum representative of PAIS (*C* = 1 M) prepared under these conditions is proposed in Figure A1. The results exploitation clearly shows that under moderate shearing rate our PAIS is almost made of cyclic macromolecular species with low molecular weight.

If the initial concentration of isosorbide is increased to 2 M, the MALDI-TOF spectrum characteristic of the corresponding polyacetal obtained in mild stirring conditions contains peaks specific of both kinds of macromolecular populations (Figure A2). However, the proportion of cyclic chains is still prevalent.

For higher monomeric concentration, the mixing operation was found to be unsatisfactory because of the immobilization of the magnetic stirring during the polymerization step. Other changes were made to the synthesis methodology presented in this paper in order to try to increase the polymer molar masses. A first trial consisted in dripping the methylene chloride (in excess) to isosorbide in DMSO instead of the one-step addition as previously described. In this case, the temperature increase during the reaction was observed to be more limited. But, the reaction yield was reduced to 30% even with the use of high speed mixing. The corresponding MALDI-TOF spectrum shows that the synthesized polyacetal is mostly under linear architecture with molar masses lower than 3300 g/mol. Some cyclic chains are detected but remain in low molar range (Figure A3).

In another exploratory pathway, the reaction between methylene chloride and isosorbide was performed in stoichiometric proportions. Such option did not increase the PAIS molar mass since a maximum value of 3000 g/mol was registered (Figure A4). However, this chemical route led to the exclusive production of linear species but with a low reaction yield (25%).
